# The Role of Active-Site Plasticity in Damaged-Nucleotide Recognition by Human Apurinic/Apyrimidinic Endonuclease APE1

**DOI:** 10.3390/molecules25173940

**Published:** 2020-08-28

**Authors:** Anatoly A. Bulygin, Alexandra A. Kuznetsova, Yuri N. Vorobjev, Olga S. Fedorova, Nikita A. Kuznetsov

**Affiliations:** 1 Institute of Chemical Biology and Fundamental Medicine, Lavrentyev Ave. 8, 630090 Novosibirsk, Russia; skytolya@ya.ru (A.A.B.); sandra-k@niboch.nsc.ru (A.A.K.); ynvorob@niboch.nsc.ru (Y.N.V.); 2 Department of Natural Sciences, Novosibirsk State University, Pirogova St. 2, 630090 Novosibirsk, Russia

**Keywords:** base excision repair, AP endonuclease, conformational dynamics, active site plasticity, apurinic/apyrimidinic site, 5,6-dihydrouridine

## Abstract

Human apurinic/apyrimidinic (AP) endonuclease APE1 hydrolyzes phosphodiester bonds on the 5′ side of an AP-site, and some damaged nucleotides such as 1,N6-ethenoadenosine (εA), α-adenosine (αA), and 5,6-dihydrouridine (DHU). To investigate the mechanism behind the broad substrate specificity of APE1, we analyzed pre-steady-state kinetics of conformational changes in DNA and the enzyme during DNA binding and damage recognition. Molecular dynamics simulations of APE1 complexes with one of damaged DNA duplexes containing εA, αA, DHU, or an F-site (a stable analog of an AP-site) revealed the involvement of residues Asn229, Thr233, and Glu236 in the mechanism of DNA lesion recognition. The results suggested that processing of an AP-site proceeds faster in comparison with nucleotide incision repair substrates because eversion of a small abasic site and its insertion into the active site do not include any unfavorable interactions, whereas the insertion of any target nucleotide containing a damaged base into the APE1 active site is sterically hindered. Destabilization of the α-helix containing Thr233 and Glu236 via a loss of the interaction between these residues increased the plasticity of the damaged-nucleotide binding pocket and the ability to accommodate structurally different damaged nucleotides. Nonetheless, the optimal location of εA or αA in the binding pocket does not correspond to the optimal conformation of catalytic amino acid residues, thereby significantly decreasing the cleavage efficacy for these substrates.

## 1. Introduction

Apurinic/apyrimidinic sites (AP-sites) are regarded as common lesions that occur in DNA spontaneously or owing to the hydrolysis of *N*-glycosidic linkages by DNA glycosylases [1,2]. It has been estimated that >10,000 AP-sites form in every mammalian cell every day and have both mutagenic and cytotoxic effects [3,4,5]. The major enzyme of base excision repair (BER), human APE1 (AP endonuclease), initiates the process of removal of AP-sites from the genome [6,7]. It is thought that the key function of this enzyme is phosphodiester bond hydrolysis on the 5′ side of an AP-site in DNA, thereby causing the cleavage of the deoxyribose-phosphate backbone and forming termini carrying a 2′-deoxyribose-5′-phosphate and 3′-hydroxyl group [8]. On the other hand, regarding substrates, it has been shown that this enzyme can recognize not only various AP-sites but also some types of damaged bases, for example, oxidatively damaged pyrimidines [9], bulky photoproducts [10], benzene-derived DNA adducts [11], etheno-derivatives of DNA bases [12,13], α-anomers of 2′-deoxynucleosides [14], and 2′-deoxyuridine [15]. In addition, the APE1 enzyme has 3′-phosphodiesterase, 3′-5′-exonuclease, and 3′-phosphatase activities [16,17].

X-ray crystallography of human APE1 when free [18,19,20] or in various complexes with DNA [21,22,23,24] has revealed that APE1 induces DNA bending and eversion of an AP-site from the double helix into the enzyme’s active site. The structural findings suggest that the rigid core of the APE1 protein causes conformational distortions of DNA because of the formation of specific contacts that are needed for a catalytically competent state. Amino acid residues Arg73, Ala74, and Lys78 come into contact with three successive DNA phosphates in the opposite strand, 5′ to the AP-site. Met270 is incorporated into the DNA minor groove, thereby displacing the nucleobase opposite the AP-site. Gly127 and Tyr128 broaden and span the DNA minor groove. Amino acid residue Arg177 is inserted through the major groove of DNA and enters into a hydrogen bond with the AP-site 3′-phosphate. The set of DNA–enzyme contacts makes the extrahelix conformation of the abasic site stable, coordinates the active-site scissile 5′-phosphate group, and results in effective enzymatic hydrolysis of the phosphodiester linkage between nucleotides. In spite of good characterization of crystal structures of human APE1 in complex with a DNA substrate containing a stable analog of a natural AP-site devoid of the OH group on the C1′ atom of ribose (i.e., an F-site) or a cleaved product (DNA), currently, there are no documented structures of an APE1 complex with substrate DNA carrying a lesion (damaged base).

In our previous study [25], an analysis of conformational dynamics during an interaction of this enzyme with DNA containing 1,N6-ethenoadenosine (εA), α-adenosine (αA), 5,6-dihydrouridine (DHU), or an F-site revealed that the DNA distortions induced by enzyme binding and initial-complex formation depend on the nature of the damaged nucleotide. It has been suggested that the key factor responsible for the substrate specificity of APE1 is the ability of a damaged nucleotide to get everted from a double helix and to get inserted into the damaged-nucleotide binding pocket. Nevertheless, it should be mentioned that in addition to the eversion of a target nucleotide from the substrate structure, its insertion into the active site should also be taken into account in such studies. Indeed, on the basis of literature data, we can compare APE1 activities on various damaged DNA duplexes: the recognition and cleavage of an F-site in a complementary duplex proceeds during a 1 s period [26,27,28], whereas the duration of recognition of such damaged nucleotides as εA, αA, or DHU is much longer and takes up to 1000 s [25,29,30]. Moreover, native nucleotides are processed in the 3′-5′ exonuclease reaction within 100–1000 s [31], but endoribonuclease cleavage of native ribonucleotides takes place much more slowly, up to hours [32]. Overall, it could be suggested that during the eversion of a small abasic site and its insertion into the active site of the enzyme, the deoxyribose residue does not engage in any unfavorable interactions. On the other hand, insertion of any kind of target nucleotide bearing a native or damaged base into the APE1 active site is far slower than abasic-nucleotide insertion owing to a steric hindrance. Consequently, the main objective of this study was to clarify the major steps in the mechanism underlying DNA–protein interaction that enable specific recognition of structurally varied damaged nucleotides αA, εA, or DHU as compared to an F-site. We analyzed the pre-steady-state kinetics of conformational alterations of APE1 and certain DNA substrates in the course of DNA binding. Changes in fluorescence intensity of tryptophan residues were recorded to characterize conformational transitions in the protein molecule. Fluorescently labeled DNA duplexes containing a damaged nucleotide and a FRET pair of dyes made it possible to detect conformational changes in DNA that were induced by the enzyme. Molecular dynamics (MD) simulations of enzyme–substrate complexes revealed that destabilization of the α-helix containing Thr233 and Glu236 via a loss of the interaction between these residues increases the plasticity of the damaged-nucleotide binding pocket and the ability to accommodate structurally different damaged nucleotides. Moreover, the obtained data allow us to conclude that the efficacy of cleavage of different substrates is related to the coherence of the optimal location of a damaged base in the enzyme’s binding pocket and optimal distances between catalytic amino acid residues and the scissile phosphate group.

## 2. Results and Discussion

### 2.1. Relative Cleavage Activity of APE1

PAGE analysis of product accumulation in the course of the interaction of APE1 with every X-substrate (X = αA, DHU, F-site, or εA as damage) was performed first. Direct registration of product formation helped to rank the efficacy of DNA cleavage as follows: F-site > DHU >> αA > εA, in line with other studies [25,30,33].

### 2.2. Effects of Mg^2+^ Ions on DNA Binding and Catalysis

**Figure 1:**
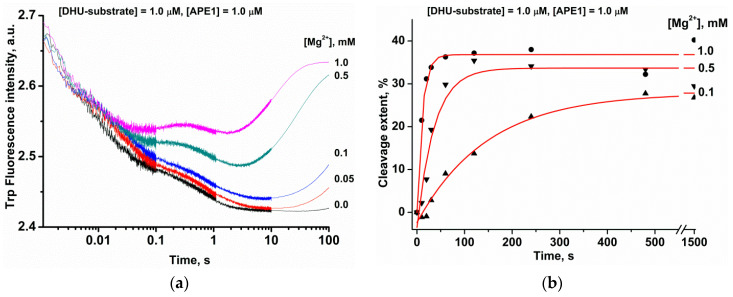
The influence of Mg^2+^ ions on the APE1 interaction with the DHU-substrate. (**a**) Stopped-flow kinetic traces of Trp fluorescence. The first syringe contained 1.0 μM APE1 that was incubated with different concentrations of MgCl_2_. The second syringe held 1.0 μM DHU-substrate and an equivalent amount of MgCl_2_. Final MgCl_2_ concentrations are indicated beside the kinetic trace. (**b**) Reaction product accumulation as evidenced by PAGE.

The DHU-substrate containing the smallest damaged pyrimidine base is cleaved with greater efficiency than αA and εA; therefore, this substrate was chosen for further detailed analysis of the substrate-binding process. It should be mentioned that the activity of APE1 depends on the concentration of Mg^2+^ [14,34]. Typically, the AP-site cleavage activity is tested in the presence of ≥5 mM Mg^2+^ [14,35,36,37,38], but the cleavage of DNA bearing a damaged base is tested at a low concentration of Mg^2+^, within the 0.01–0.5 mM range [12,14,35,36,39]. Consequently, to verify the efficiency of APE1 binding to the DHU-containing DNA duplex at various Mg^2+^ levels, the kinetics of substrate–enzyme complex formation and of the catalysis were investigated by the stopped-flow method (Figure 1a). Without Mg^2+^ ions, only a decreased phase of Trp fluorescence intensity was recorded, reflecting the formation of an enzyme–substrate complex. With an increase in the concentration of Mg^2+^, this process accelerated, and the growth phase appeared at later reaction time points, indicating the progression of a catalytic reaction and subsequent dissociation of the enzyme–product complex. Indeed, the onset of the phase of the increase in intensity at late time points is consistent with the onset of the product accumulation revealed by PAGE (Figure 1b).

### 2.3. Analysis of DNA Conformational Changes in the Course of Interaction with APE1

**Figure 2:**
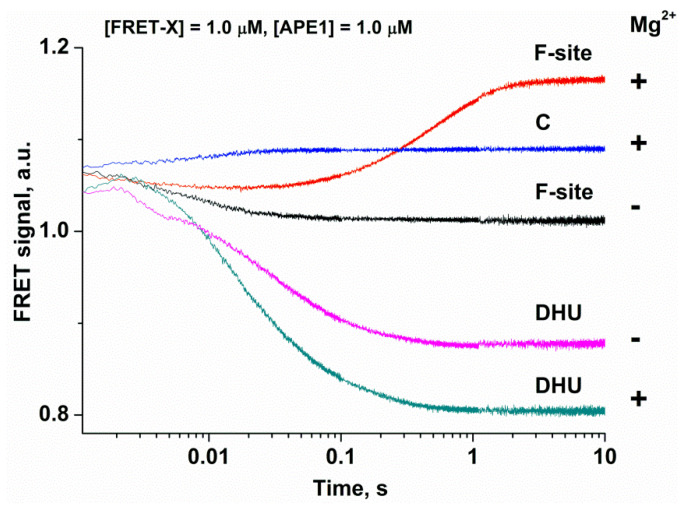
Changes in the FRET signal during the interaction of APE1 with each FRET-X-substrate in the absence “−” or presence “+” of 5.0 mM Mg^2+^. FRET traces of the interaction of APE1 with the FRET-F- and FRET-DHU-substrates in the absence of Mg^2+^ have been reported earlier [25].

To analyze conformational changes in DNA during the interaction with APE1, we used FRET-X-substrates, where X = C, F-site, or DHU (Figure 2). In the FRET experiments, to prevent the possible effects associated with unsaturation of the active site of the enzyme by Mg^2+^, we employed a high MgCl_2_ concentration (5.0 mM) in reaction buffer. These conditions clearly illustrated the difference between the DNA-binding process alone (absence of Mg^2+^ in the active site) and DNA binding with a subsequent catalytic step (the active site saturated by the Mg^2+^ ion). Duplex FRET-C served as a control for conformational changes occurring during the formation of a nonspecific complex. As presented in Figure 2, the interaction of APE1 with the FRET-C duplex (blue trace) leads to a slight increase in the FRET signal within the period up to time point 0.1 s. When a catalytically inactive form of apo-APE1 interacted with the FRET-F-substrate in the absence of Mg^2+^ ions (black trace) and catalysis was blocked, only the phase of the FRET signal decrease was recorded. The decrease in the FRET signal indicates that FAM and the BHQ1 quencher became spatially close to each other, and this event may be associated with bending of the DNA backbone. Otherwise, in the presence of Mg^2+^ ions (red trace), in the region of the kinetic curves up to 0.02 s, a rapid decrease in the FRET signal takes place, reflecting the formation of the enzyme–substrate complex, followed by a growth phase between time points 0.05 and 5 s. The increase in the FRET signal is associated with an increase in the distance between FAM and the BHQ1 quencher, and this event is possible after the catalytic step and dissociation of the enzyme–product complex. The visually obvious decrease in FRET signal amplitude for FRET-F-substrate in the presence of Mg^2+^ ions is related to the overlap of the complex formation stage with catalytic stage and dissociation of the enzyme–product complex, thereby leading to an increase in the FRET signal.

As depicted in Figure 2, the interaction of APE1 with the FRET-DHU-substrate in the absence of Mg^2+^ ions induces a decrease in the FRET signal up to time point 1 s, thus reflecting the formation of the complex. These data revealed that the bending of DNA containing DHU is ~10-fold slower in comparison with an abasic site (1 s and 0.1 s, respectively). By contrast, in the presence of Mg^2+^ ions, the growth phase characterizing the catalytic stage was not detectable, as in the case of the F-site, indicating that the duplex bending alone is not a sufficient process for the formation of the catalytically competent state of the active site.

### 2.4. MD Simulations

**Figure 3:**
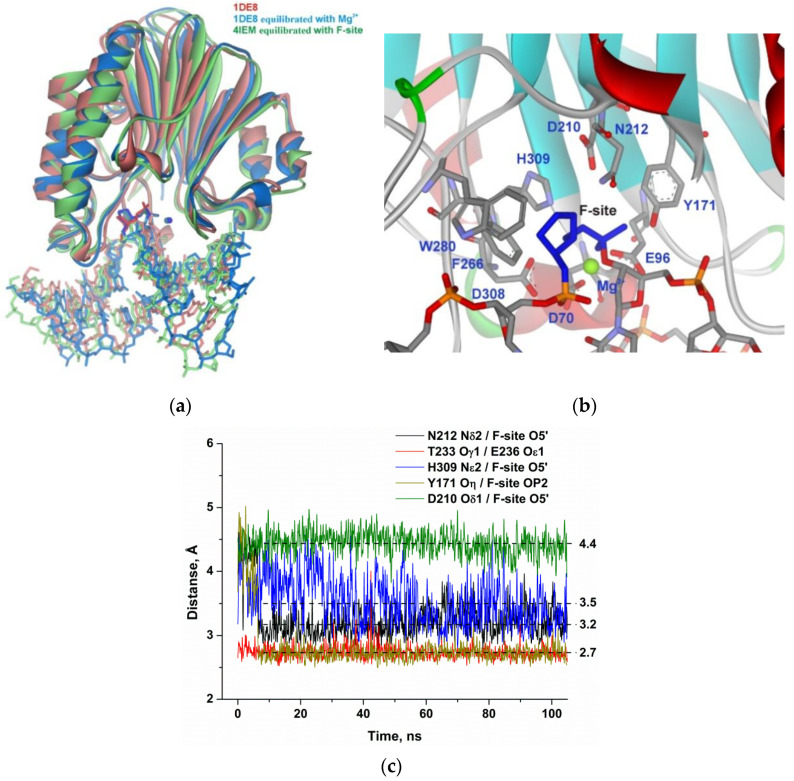
Molecular dynamics (MD) structure and trajectories of the APE1 complex with DNA containing the F-site. (**a**) Comparison of the 1DE8 X-ray structure (red) with equilibrated structures of the complex of APE1 with F-site-containing DNA and a Mg^2+^ ion derived from 1DE8 (blue) or 4IEM (green). The F-site and Mg^2+^ ion are highlighted in the same color as proteins. (**b**) The structure of APE1′s binding pocket adapted to an F-site. The F-site and key amino acid residues of the damaged-nucleotide–binding pocket are shown. (**c**) Changes in distances between catalytic amino acid residues and the scissile phosphate group in the dynamic trajectories for the complexes of APE1 with DNA containing the F-site.

Structures of the complex of APE1 with F-site-containing DNA and a Mg^2+^ ion (retrieved from X-ray structures 1DE8 and 4IEM) were equilibrated within 10 ns to stabilize total potential energy of the complex. As shown in Figure 3a, the equilibrated structures of the complex of APE1 with F-site-containing DNA and a Mg^2+^ ion have very similar positions of the F-site located in the active site; the same is true for the Mg^2+^ ion. Equilibrium 100 ns MD trajectories were generated for both APE1–DNA complexes and did not reveal significant differences in the final structures.

Specific distances between atoms of the F-site and active-site amino acid residues did not significantly change throughout the MD trajectory (Figure 3b). The phosphate group of the F-site forms stable H-bonds with Asn212 and Tyr171 and engages in a strong interaction with the Mg^2+^ ion (Figure 3c). The weak unstable contact of the bridging oxygen atom of the scissile phosphate group with His309 features the average distance of 3.5 Å during the final 50 ns of the MD trajectory. The distance between the carboxyl group of the catalytic Asp210 residue and O5′ of the F-site was also very stable. The distances observed in the complex of the enzyme with F-site-containing DNA were used as reference values of the catalytically competent state of the active site for comparisons with other substrates.

On the basis of the equilibrated structure of the complex of APE1 with F-site-containing DNA, initial structures of the APE1 complex with DNA containing DHU, αA, or εA were obtained. They were equilibrated also within 10 ns to stabilize the complex’s total potential energy. Next, equilibrium MD trajectories (100–180 ns) were obtained at 300 K (room temperature) for every complex of APE1 with DNA.

**Figure 4:**
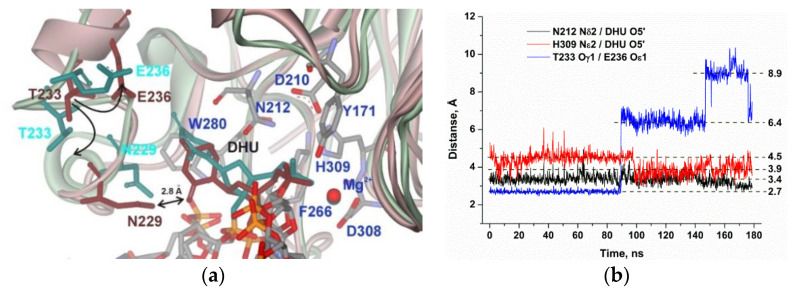
MD structures and trajectories of the APE1 complex with DNA containing DHU. (**a**) The structure of APE1′s binding pocket adapted to DHU blocked by Asn229 (reddish) or transformed to the catalytic state (greenish). DHU, Asn229, Thr233, and Glu236 are highlighted in brown (blocked state) or dark cyan (catalytic state). Key amino acid residues of the damaged-base–binding pocket are shown. (**b**) Changes in distances between catalytic amino acid residues and the scissile phosphate group in the dynamic trajectories of the complexes of APE1 with DNA containing DHU.

The productive MD simulation of the structure of the APE1 complex with DHU after energy optimization and simulated annealing revealed two stable states of the damaged nucleotide in the active site (Figure 4a). In the initial part of the MD trajectory (Figure 4b), the Asn229 residue managed to form an H-bond with O2 of DHU, thus leading to the stabilization of this state. Then, Asn229 came into contact with Asn226 and unblocked the DHU base. Moreover, analysis of the MD trajectories indicated that the loss of the direct Asn229–DHU contact is accompanied by the loss of the H-bond between residues Thr233 and Glu236, thus resulting in significant loop reorganization (Figure 4a), thereby allowing the DHU base to acquire mobility, shifting the whole nucleotide deeper into the active site of the enzyme. Interestingly, no steric hindrance is caused by the amino acid backbone of the loop residues, but rather the loop reorganization leads to an Asn229 shift together with the amino acid backbone. This phenomenon is associated with the rotation of the Asn229 side chain and the loss of a direct contact with O2 of the DHU base. This relocation of DHU leads to a decrease in the average distance between His309 and the scissile phosphate group from 4.5 to 3.9 Å, which enables the catalytic state of the active site.

**Figure 5:**
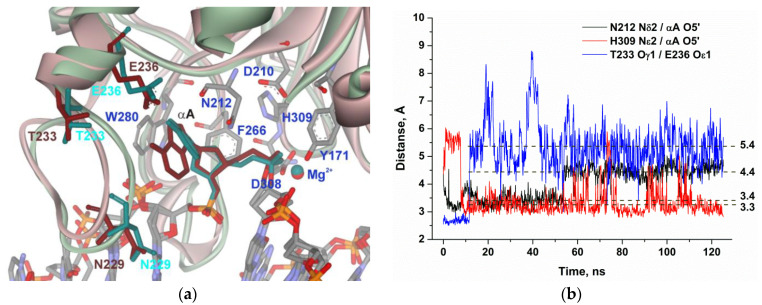
MD structures and trajectories of the APE1 complex with DNA containing αA. (**a**) The structure of APE1′s binding pocket adapted to the initial state of αA (reddish) or in the disrupted final state (greenish). αA, Asn229, Thr233, and Glu236 are highlighted in brown (initial state) or dark cyan (disrupted state). Key amino acid residues of the damaged-base–binding pocket are shown. (**b**) Changes in distances between catalytic amino acid residues and the scissile phosphate group in the dynamic trajectories for the complexes of APE1 with DNA containing αA.

It is noteworthy that, in the case of αA (Figure 5), the decrease in the distance between His309 and the scissile phosphate group was also accompanied with a loss of the interaction between Thr233 and Glu236 and destabilization of the loop. Nevertheless, the subsequent turn and shift of the αA base during MD simulation cause an increase in the distance from another catalytic residue, Asn212, from 3.4 to 4.4 Å, supporting the disruption of the catalytic conformation. These data indicate that the optimal location of the αA base in the enzyme’s binding pocket does not correspond to an optimal conformation of catalytic amino acid residue Asn212, thereby correlating with experimental data on reduced efficiency of cleavage of the αA-substrate in comparison with the DHU-substrate. These findings also suggest that conformational instability of the loop region may be important for the recognition of a damaged nucleotide.

**Figure 6:**
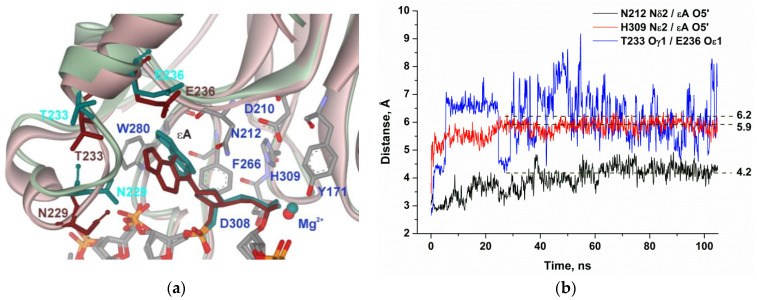
MD structures and trajectories of the APE1 complex with DNA containing εA. (**a**) The structure of APE1′s binding pocket adapted to the initial state of εA (reddish) or in the disrupted final state (greenish). εA, Asn229, Thr233, and Glu236 are highlighted in dark cyan (initial state) or brown (disrupted state). Key amino acid residues of the damaged-base–binding pocket are presented. (**b**) Changes in distances between catalytic amino acid residues and the scissile phosphate group in the dynamic trajectories for the complexes of APE1 with DNA containing εA.

The MD simulation of the complex of APE1 with DNA containing εA (Figure 6) also uncovered the loss of the direct Thr233–Glu236 interaction in the initial part of the trajectory. Moreover, distances between residues Asn212 and His309 and the phosphate group are growing during the initial 30–50 ns of the simulation and stabilize at 4.2 and 5.9 Å, respectively, which supports the full disruption of catalytic-network contacts.

**Figure 7:**
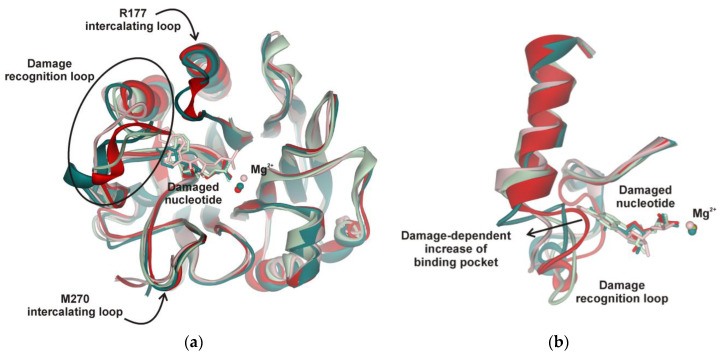
(**a**) Superposition of the MD structures of the APE1 complex with DNA containing an F-site (red), DHU (dark green), εA (reddish), or αA (green). Damaged nucleotides inserted into APE1′s binding pocket. Stable positions of intercalating loops designated as Arg177 and Met270 are shown. (**b**) Close-up view of the damage recognition loop adapted to different damaged nucleotides.

A comparison of all the structures revealed that the Mg^2+^ ion engages in a stable interaction with a nonbridged oxygen atom of the scissile phosphate group. Indeed, superposition of the MD structures at minimal total potential energy (Figure 7a) showed that the Mg^2+^ ion is moved to the active site together with the phosphate group of the damaged nucleotide. Therefore, the experimentally determined dependence of the enzymatic activity on the concentration of Mg^2+^ most likely is related to saturation of the active site by Mg^2+^, which is strongly needed to coordinate the scissile phosphate group. The overlap of all structures indicated that both functionally important loops containing Arg177 and Met270, which intercalate into DNA to stabilize the extrahelical state of the damaged nucleotide, are very stable in the complexes with all the tested damaged nucleotides. Otherwise, the loop containing Asn229/Thr233/Glu236 underwent significant damage-dependent reorganization, indicating the important role of this loop in the recognition of the damaged nucleotide (Figure 7a). Indeed, the close-up view illustrates the plasticity of this loop depending on the size of the damaged nucleotide (Figure 7b).

Taken together, the obtained data mean that the movement of the recognition loop is associated with optimal accommodation of the damaged base in the pocket of the active site. For instance, in the case of DHU, two stable conformational states exist, in which DHU is blocked by Asn229 or fully inserted into the active site. The hydrolysis of the phosphodiester bond is possible only in the second state due to the formation of optimal distances to catalytic residues Asn212 and His309. Experimental data revealed that DHU-substrate cleavage (Figure 1b) reaches a plateau at ~30%, indicating the saturation of the second catalytic state at this level and suggesting that the transition between these states is associated with energy costs and proceeds slowly. On the other hand, the cleavage of αA- and εA-substrates is much less efficient because the optimal position of these bases in the binding pocked does not correspond to optimal distances between the scissile phosphate group and catalytic amino acid residues. Indeed, in the case of the αA base, Asn212 moves away and cannot come into contact with the phosphate group, but in the case of the biggest base (εA), the contacts with both Asn212 and His309 are lost.

Of note, alignment of amino acid sequences of AP endonucleases from *Danio rerio*, *Xenopus laevis*, and *Drosophila melanogaster* indicates that Thr233 and Glu236 are fully conserved among these species, whereas Asn229 is substituted by Thr only in the AP endonuclease from *Xenopus laevis*. Therefore, our data on active-site plasticity seem to reflect a common feature of AP endonucleases of the APE1 structural family. Moreover, because of the X-ray data analysis, it is known that AP endonuclease APE1 has a rigid core that slightly differs between the free enzyme and the enzyme complexed with an abasic DNA. Because there are no structural data on the complexes of APE1 with other types of damaged nucleotides, we assumed that this rigidity is a common feature of APE1 proteins. The results obtained in the present study indicate that during recognition of various damaged nucleotides, the DNA-binding site of APE1 must undergo conformational changes to accommodate the nucleotides containing the damaged base. Therefore, these findings constitute the evidence of high plasticity of the damaged-nucleotide–binding pocket of APE1.

## 3. Materials and Methods

### 3.1. Protein Expression and Purification

Wild-type APE1 was expressed and purified as described previously [40,41]. The protein concentration was measured by the Bradford method [42]; the stock solution was stored at −20 °C.

### 3.2. Oligodeoxynucleotides (ODNs)

Sequences of the ODNs employed in this study are given in Table 1. These ODNs were prepared by widely accepted phosphoramidite methods by means of an ASM-700 synthesizer (BIOSSET Ltd., Novosibirsk, Russia) from phosphoramidites that were bought from Glen Research (Sterling, VA, USA). α-2′-Deoxyadenosine phosphoramidite was purchased from ChemGenes Corp. (Wilmington, MA, USA). The synthesized ODNs were separated from solid support by means of ammonium hydroxide in keeping with the manufacturer’s instructions. The deprotected ODNs were purified via high-performance liquid chromatography. Concentrations of the ODNs were computed from A_260_. ODN duplexes were generated via annealing of modified and complementary strands in a molar ratio of 1:1.

### 3.3. PAGE (Polyacrylamide Gel Electrophoresis) and Enzymatic Analyses

The endonuclease assay was carried out in reaction buffer (50 mM Tris-HCl pH 7.5, 50 mM KCl, 1.0 mM DDT, 1.0 mM EDTA, 7% [*v*/*v*] of glycerol, and various levels of MgCl_2_ within 0.0–1.0 mM). The reaction mixture contained 1.0 μM DNA substrate and 1.0 μM APE1. To increase enzyme stability during the experimental procedures, all the experiments were conducted at 25 °C. The reactions were allowed to proceed at 25 °C and were stopped by a gel-loading dye containing 50 mM EDTA and 7 M urea and were next loaded onto a 20% polyacrylamide gel (*w*/*v*) including 7 M urea. Formation of the product and disappearance of the substrate were investigated by autoradiography and quantitated via scanning densitometry using Gel-Pro Analyzer software v.4.0 (Media Cybernetics, Rockville, MD, USA).

### 3.4. Stopped-Flow Measurements with Fluorescence Detection

These measurements were taken mostly as described before [43,44]. In short, we utilized a SX.18MV stopped-flow spectrometer (Applied Photophysics Ltd., Leatherhead, UK) equipped with an optical cell with 2 mm path length and a 150 W Xe arc lamp. The dead time of this equipment is known: 1.4 ms. Trp fluorescence was excited at 290 nm (λ_ex_) and monitored at >320 nm (λ_em_) as transmitted by the WG-320 filter (Schott, Mainz, Germany). 6-Carboxyfluorescein (FAM) residue fluorescence was excited at 494 nm and detected at >515 nm, as transmitted by the OG-515 filter (Schott, Mainz, Germany). The experiments on Trp fluorescence detection were carried out at 25 °C with catalytically active APE1 in a buffer composed of 50 mM Tris-HCl pH 7.5, 1.0 mM DTT, 50 mM KCl, 7% of glycerol (*v*/*v*), and various levels of MgCl_2_ within 0.0–1.0 mM. Detection of the process of DNA binding using a FRET signal was performed at 25 °C with Mg^2+^-free catalytically inactive apo-APE1 in a buffer composed of 50 mM Tris-HCl pH 7.5, 1.0 mM EDTA, 1.0 mM DTT, 50 mM KCl, and 7% of glycerol (*v*/*v).* In all the experiments, when the concentration of Mg^2+^ was declared to be zero, first of all, the catalytic activity of APE1 was abrogated by the treatment with EDTA. For this purpose, the solution of APE1 was incubated with 1.0 mM EDTA for 5 min to chelate any divalent metal ions and to obtain catalytically inactive apo-APE1. The reaction solution was supplemented with a certain concentration of MgCl_2_ when required. Of note, our previous data [34] have revealed that supplementation of the solution of apo-APE1 and 1.0 mM EDTA by MgCl_2_ (even at 0.05 mM) restores catalytic activity to some extent with subsequent saturation of the enzyme by the Mg^2+^ ion with the increasing concentration of MgCl_2_. Therefore, in the present report, we used the same design of experiments.

APE1 was added into one of the syringes of the instrument and was mixed rapidly in a reaction chamber with a DNA substrate that came from the other syringe. Concentrations of APE1 and the DNA substrate were 1.0 μM in all the assays. The reported concentrations of reactants correspond to the concentrations in the reaction chamber upon mixing. As a rule, each trace depicted in the figures is the mean of ≥4 fluorescent traces acquired in individual experiments.

Protein conformational alterations were examined via changes in fluorescence intensity of Trp. FRET-X-substrates modified at 5′ ends with the dye–quencher couple FAM/BHQ1 (Table 1) were analyzed by Förster resonance energy transfer (FRET) measurements. The latter detected changes in the distance between the quencher and dye during DNA helix distortion in the course of APE1–DNA complex formation. In figures, for clarity, the curves were moved apart by hand. This approach does not influence the results of fitting: background fluorescence is fitted for each curve individually.

### 3.5. MD Simulations

Molecular modeling of the complex of APE1 with each DNA duplex containing DHU, εA, αA, or an F-site (Table 1) involved the following stages: (i) building up the initial atomic structure of the complex, (ii) atomic-structure refinement for the complex through simulated annealing and energy optimization, (iii) productive MD modeling of every complex and collection of a representative series of instant structures throughout an equilibrated MD trajectory.

A comparison of several X-ray crystal structures was performed to choose an initial structure for the simulations of APE1 with damaged DNA. We analyzed the 2.95 Å resolution structure (Protein Data Bank [PDB] ID 1DE8) of the APE1 complex with DNA containing an F-site without a Mg^2+^ ion [21], the 1.63 Å resolution structure (PDB ID 5DFI) of the APE1 complex with DNA containing 2′-O-methyl phosphorothioate backbone modification 5′ to an F-site without a Mg^2+^ ion [24], and 2.39 Å resolution structure (PDB ID 4IEM) of the APE1 complex with product DNA with a Mg^2+^ ion [23]. A comparison of the 1DE8 and 5DFI structures shows a close common overlap of protein and DNA molecules. Nevertheless, in the 5DFI structure, the phosphorothioate backbone modification has two isomers, *S*p and *R*p. Even though both isomers are everted into the active site, the *R*p isomer is shifted away from the active site by 2.1 Å. This observation indicates that the modification in question has some influence on the structure of DNA bound to the active site of the enzyme; therefore, this structure was not appropriate for MD simulation. Then, for the 1DE8 structure, the Mg^2+^ ion was added, and for the 4IEM structure, product DNA with a hydrolyzed phosphodiester bond was replaced by an F-site. Both modified structures were refined by energy optimization and simulated annealing. The final structures of the complex of APE1 with F-site-containing DNA and a Mg^2+^ ion were very similar and were chosen for productive modeling of the MD of APE1 complexes with each tested damaged nucleotide.

The simulations were performed by means of GROMACS 2019.5 [45] and the AMBER ff99SB-ILDN force field [46]. Hydrogen atoms were placed onto the structure using *pdb2gmx,* and the solvent consisted of TIP3P [47] water in periodic dodecahedron boxes containing a protein–DNA complex and at least a 1 nm layer of the solvent. Solvent molecules were replaced with counterions until the system was neutralized. Simulations were performed under 300 K with protonated histidine residues. The Verlet cutoff approach [48] was employed with a cutoff of 1.2 nm for both van der Waals and electrostatic interactions, whereas LINCS was utilized to constrain bonds [49]. Electrostatic interactions were computed in PME [50]. The solvated systems were minimized next (through steepest-descent minimization). After that, the systems were equilibrated over two stages with positional restraints on DNA and protein atoms. First of all, the systems were equilibrated for 400 ps in the NVT ensemble with subsequent equilibration for additional 400 ps in the NPT ensemble. At last, production dynamics were realized with a 2 fs time step in the NPT ensemble, and every 10 ps, the coordinates were saved. All simulations were conducted for at least 100 ns. To prevent DNA despiralization at the ends, 16 kJ/(mol·nm^2^) restraints on the terminal nucleotide atoms were applied [51].

## Figures and Tables

**Table 1:** DNA sequences and structures of modified nucleotides *.

Shorthand	Sequences of DNA Duplexes
X-substrate X = F-site, DHU, εA, or αA	5′-TCTCTCXCCTTCC-3′ 3′-AGAGAGGGGAAGG-5′
FRET-X-substrate Y = F-site, DHU, εA, αA, or C	5′-FAM-GCTCAXGTACAGAGCTG-3′ 3′-CGAGTGCATGTCTCGAC-BHQ1-5′

* FAM is 6-carboxyfluorescein, BHQ1 is black hole quencher.

## Abbreviations

AP-site: apurinic/apyrimidinic site;
APE1: human AP endonuclease;
ODN: oligodeoxyribonucleotide.
